# Hemophagocytic lymphohistiocytosis with leukoencephalopathy in a patient with dermatomyositis accompanied with peripheral T-cell lymphoma: a case report

**DOI:** 10.1186/s13256-016-0986-4

**Published:** 2016-08-02

**Authors:** Satoru Teshigawara, Yoshinori Katada, Yuichi Maeda, Maiko Yoshimura, Eriko Kudo-Tanaka, Soichiro Tsuji, Yoshinori Harada, Masato Matsushita, Shiro Ohshima, Kotaro Watanabe, Takahiro Kumode, Yoshihiko Hoshida, Yukihiko Saeki

**Affiliations:** 1 Department of Rheumatology and Allergology, NHO Osaka-Minami Medical Center, 2-1 Kidohigashi-machi, Kawachinagano, Osaka 586-8521 Japan; 2 Department of Stroke Center, NHO Osaka-Minami Medical Center, 2-1 Kidohigashi-machi, Kawachinagano, Osaka 586-8521 Japan; 3 Division of Hematology and Rheumatology, Kinki University Faculty of Medicine, 377-2 Ono-higashi, Osaka-sayama, Osaka 589-8511 Japan; 4 Department of Pathology, NHO Osaka-Minami Medical Center, 2-1 Kidohigashi-machi, Kawachinagano, Osaka 586-8521 Japan; 5 Department of Clinical Research, NHO Osaka-Minami Medical Center, 2-1 Kidohigashi-machi, Kawachinagano, Osaka 586-8521 Japan; 6 Department of General Internal Medicine, Graduate Medical Education and Clinical Investigation, Sakai City Medical Center, Ebaraji-cho 1-1-1, West Ward, Sakai, Osaka 593-8304 Japan

**Keywords:** Dermatomyositis, Hemophagocytic lymphohistiocytosis, Leukoencephalopathy, HLH-2004 protocol, Peripheral T-cell lymphoma

## Abstract

**Background:**

Hemophagocytic lymphohistiocytosis associated with autoimmune diseases is seen in patients with systemic juvenile idiopathic arthritis, adult-onset Still’s disease, and systemic lupus erythematosus, whereas it is rarely seen in patients with dermatomyositis. In addition, central nervous system involvement with dermatomyositis is rare. To the best of our knowledge, this is the first case of hemophagocytic lymphohistiocytosis complicated by leukoencephalopathy in a patient with dermatomyositis accompanied with peripheral T-cell lymphoma.

**Case presentation:**

A 17-year-old Asian male adolescent with dermatomyositis and hemophagocytic lymphohistiocytosis that were controlled with corticosteroid therapy presented to our hospital with high fever and altered consciousness. Brain magnetic resonance imaging revealed multiple cerebral lesions. We diagnosed the central nervous system lesions as leukoencephalopathy secondary to dermatomyositis and hemophagocytic lymphohistiocytosis. Because corticosteroid and cyclophosphamide pulse therapy was ineffective, he was treated with a modified hemophagocytic lymphohistiocytosis-2004 protocol, which resulted in the disappearance of the lesions of his central nervous system.

**Conclusions:**

Our findings suggest that the hemophagocytic lymphohistiocytosis-2004 protocol including etoposide should be initiated immediately in patients with hemophagocytic lymphohistiocytosis who respond poorly to treatment for the underlying disease. Moreover, irrespective of the underlying disease, patients with hemophagocytic lymphohistiocytosis with central nervous system lesions might require bone marrow transplantation.

## Background

Hemophagocytic lymphohistiocytosis (HLH) is a life-threatening hyperinflammatory syndrome associated with a variety of underlying conditions. The hereditary form of HLH is caused by defects in the transport, processing, and function of cytotoxic granules in natural killer cells and cytotoxic T lymphocytes, and it can manifest in childhood or adulthood. The acquired forms of HLH are caused by infectious diseases, autoinflammatory and autoimmune diseases, malignancies, and acquired immune deficiency [1]. HLH associated with autoimmune diseases is seen in patients with systemic juvenile idiopathic arthritis, adult-onset Still’s disease, and systemic lupus erythematosus, whereas it is rarely seen in patients with dermatomyositis (DM) [2]. HLH is usually treated with intensive immunosuppressive therapy, including immunomodulatory and immunosuppressive agents. The therapy aims to suppress hypercytokinemia and to eliminate activated and infected cells. The protocol based on HLH-1994/HLH-2004 (with or without cyclosporine A in the first 8 weeks) is currently regarded as the standard of care [3, 4]. Patients with hereditary HLH can be cured only with hematopoietic stem cell transplantation, while patients with acquired HLH have shown improved survival when treated with reduced-intensity conditioning regimens [1].

DM is an idiopathic acute inflammatory disorder, characterized by inflammation of skeletal muscle, progressive symmetrical proximal myopathy, and classical cutaneous manifestations. The disease is associated with a connective tissue disease or a malignancy [5]. Central nervous system (CNS) involvement with DM is rare, and to the best of our knowledge, only five cases have been reported that were associated with juvenile DM, which is a rare, serious autoimmune condition of childhood involving systemic small vessel vasculopathy. It typically affects skin and muscle, but it can also involve the joints, gut, lung, heart, and other internal organs [6].

## Case presentation

**Fig. 1:**
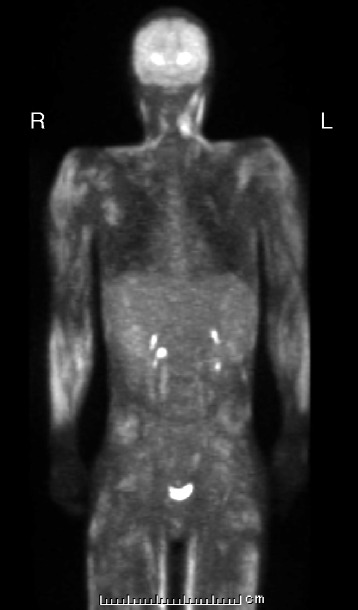
A positron emission tomography-computed tomography examination at the onset of dermatomyositis and hemophagocytic lymphohistiocytosis. No mass lesions was detected

A 17-year-old Asian male adolescent was admitted to our hospital with a chief complaint of sudden-onset fever and altered consciousness. He had a 6-month history of DM complicated by HLH controlled with corticosteroids (60 mg daily) and no obvious family, psychosocial, and genetic history. At the time of diagnosis (6 months prior to admission), heliotrope eruption was seen. A blood examination showed no evidence of myositis-specific antibody (anti-aminoacyl antibody). A muscle biopsy revealed lesions that were consistent with myositis; he met the diagnostic criteria of DM. A positron emission tomography-computed tomography (PET-CT) examination (Fig. 1), bone marrow examination, and liver biopsy had shown no malignancy; therefore, his corticosteroid dose was gradually reduced to 8 mg daily. However, he experienced intermittent leg pain, and laboratory data showed a gradual elevation of creatine kinase (CK) levels and progression of leukopenia over the preceding 6 months; therefore, his steroid therapy was increased to 30 mg daily.

**Fig. 2:**
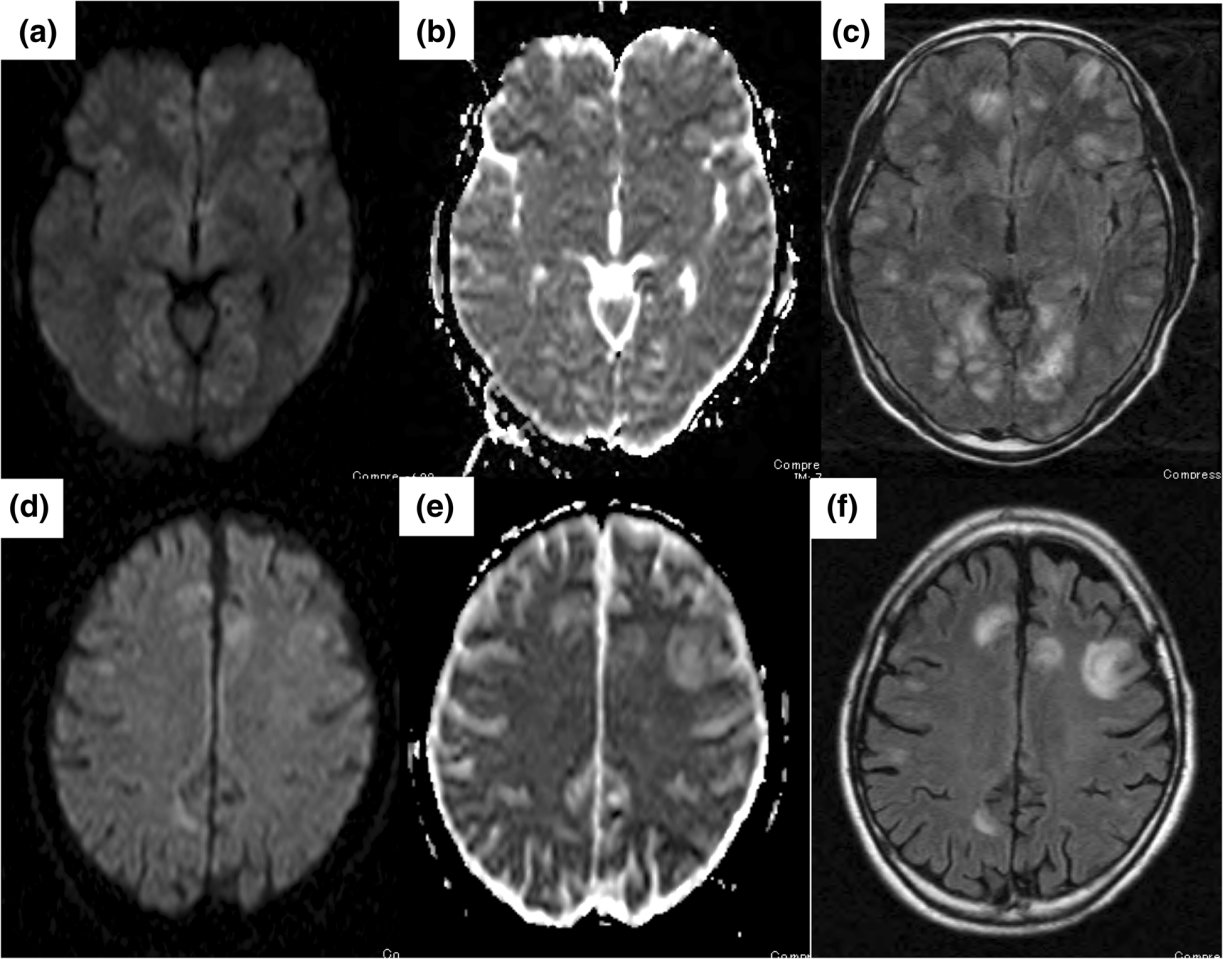
Brain magnetic resonance imaging on the day of admission and on day 261 (showing recurrence). **a–c** Images on the day of admission; **d–f** images obtained on day 261 when the patient’s central nervous system symptoms deteriorated. **a** and **d** Diffusion-weighted sequences images. **b** and **e** Apparent diffusion coefficient images. **c** and **f** Fluid-attenuated inversion recovery images. **a, b, d, e** show that central nervous system lesions did not derive from vasculitis and cerebrovascular diseases because those were not along the vessels. Hyperintensity in the cortex and subcortical white matter can be seen in **c** and **f. c** Margins of the lesion are clearly visualized. **f** Margins of the lesion are unclear

On the day of admission, he was irritable, but there were no remarkable findings on physical examination. Laboratory data showed increased levels of CK (1869 U/L), soluble interleukin-2 receptor (4120 U/mL), and ferritin (967.4 ng/mL) and decreased counts of white blood cells (3910/μL) and platelets (124,000/μL). C-reactive proteins were hardly detected (0.01 mg/dL). We suspected exacerbation of DM and HLH. Active hemophagocytosis and no malignancy were observed on bone marrow examination. Brain magnetic resonance imaging (MRI) revealed multiple bilateral subcortical lesions (Fig. 2). Systemic computed tomography (CT) did not show any evidence of interstitial pneumonia or malignancy. Cerebrospinal fluid (CSF) aspiration revealed the presence of 12 cells (nine neutrophils and 3three lymphocytes), glucose levels of 57 mg/dL, protein levels of 44 mg/dL, adenosine deaminase levels of 3.9 (>1) U/L, and interleukin-6 levels of 91.9 (>4.3) pg/mL. Polymerase chain reaction (PCR) analyses of his CSF showed no evidence of a recent infection with the Epstein–Barr virus, cytomegalovirus, varicella zoster virus, human herpes virus 6, and herpes simplex virus. We could not perform a PET-CT examination on admission because of his altered consciousness. On the basis of the MRI findings, elevation of CSF interleukin-6 levels, peripheral blood cell count, and biochemistry results, and the hemophagocytosis observed in the bone marrow, we diagnosed the CNS lesions as leukoencephalopathy secondary to DM and HLH.

**Fig. 3:**
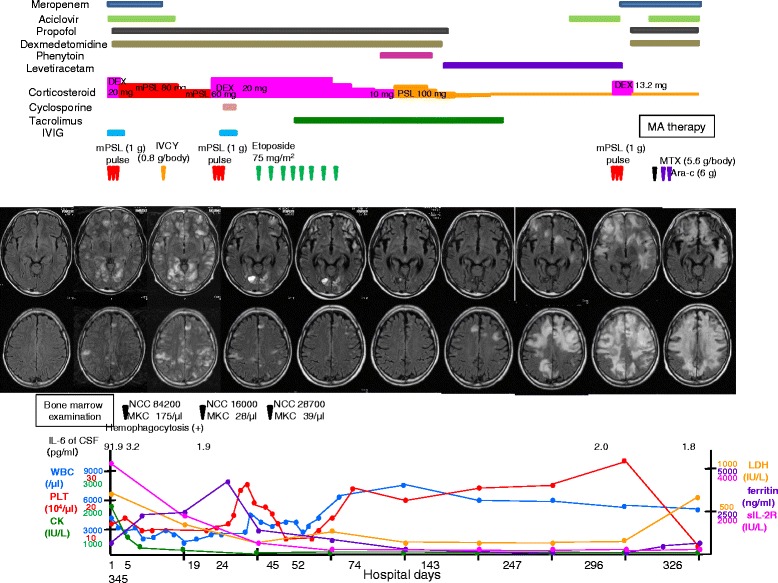
Clinical course of the patient. The patient’s central nervous system lesions disappeared completely on day 143 after treatment with methylprednisolone pulse therapy, cyclophosphamide administered intravenously, tacrolimus, and etoposide. However, he experienced a recurrence of the lesions and ultimately died of sepsis on day 348. Laboratory data show that from day 0 to day 80 his white blood cells and platelets decreased and increased. His creatine kinase levels reached a normal range at an early stage, and his soluble interleukin-2 receptor and lactate dehydrogenase levels, biomarkers of malignant lymphoma, were almost normal throughout. In the early stage, we could not distinguish his central nervous system lesions from central nervous system infectious disease owing to hemophagocytic lymphohistiocytosis with dermatomyositis; therefore, we administered meropenem and acyclovir. In the first 5 months, we administered sedatives, such as propofol and dexmedetomidine, and antiepileptic drugs, such as phenytoin and levetiracetam. *Ara-c* cytarabine, *CK* creatine kinase, *CSF* cerebrospinal fluid, *DEX* dexamethasone, *IL-6* interleukin-6, *IVCY* cyclophosphamide administered intravenously, *IVIG* intravenous immunoglobulin, *LDH* lactate dehydrogenase; *MA therapy* combination therapy with methotrexate and cytarabine, *MKC* megakaryocyte, *mPSL* methylprednisolone, *MTX* methotrexate, *NCC* nuclear cell count, *PLT* platelet, *PSL* prednisolone, *sIL-2R* soluble interleukin-2 receptor, *WBC* white blood cell

The clinical course of our patient is presented in Fig. 3. Dexamethasone (DEX) pulse therapy (20 mg daily for 3 days) and intravenous immunoglobulin therapy (400 mg/kg/day for 5 days) were initiated on the day of admission to treat his CNS lesions. Methylprednisolone (mPSL; 60 mg daily) was administered post-treatment. His CK levels improved rapidly (from 1869 U/L on the day of admission to 287 U/L on day 14); however, his bicytopenia (leukopenia and thrombopenia) remained unchanged, and his CNS lesions were still observed. Therefore, cyclophosphamide pulse therapy was added on day 8. On day 24, a second bone marrow examination revealed that his hemophagocytosis remained, for which cyclosporine was started the next day. On day 28, the 60 mg daily mPSL was changed to 20 mg daily DEX because DEX diffuses to the CSF more easily than does mPSL. In addition, as his hemophagocytosis persisted, etoposide (120 mg/body) was started on day 38 and was administered once per week between days 38 to 89 (eight times in total). On day 47, tacrolimus was administered instead of cyclosporine to suppress hemophagocytosis because both drugs have the same effects and because of a severe liver function disorder due to cyclosporine (aspartate transaminase and alanine transaminase levels increased to 318 and 917 IU/mL, respectively). Subsequently, his bicytopenia (leukopenia and thrombopenia) recovered to within normal ranges, and his CNS lesions disappeared.

He was administered sedative drugs to produce a state of calmness, to help him sleep, and to prevent acts of violence caused by his altered state of consciousness that lasted for 5 months. The medications were eventually discontinued because altered consciousness was no longer observed. However, disturbances of his higher cerebral functions remained. Five months after his initial admission, he could speak and walk like a very young child. Corticosteroid therapy was successfully tapered without a relapse of his symptoms (including fever and altered consciousness), and without changes in his laboratory and MRI findings.

Eight months after admission, a follow-up brain MRI revealed a recurrence of the CNS lesions (Fig. 2), although these recurrent lesions were different from the previous lesions, considering that there was no fever and no altered consciousness. Moreover, his laboratory findings were normal. We hypothesized that the pathology of the new CNS lesions differed from the pathology of the previous lesions and presumed that they were caused by progressive multifocal leukoencephalopathy or drug-induced (tacrolimus) leukoencephalopathy. However, a PCR analysis for the John Cunningham virus in his CSF (to diagnose progressive multifocal leukoencephalopathy) was negative, and no improvement in CNS lesions was observed when tacrolimus was discontinued.

One month after the recurrence of the CNS lesions, his abilities to walk and speak deteriorated. A MRI revealed a progression of the CNS lesions; therefore, a brain biopsy of his right frontal lobe was performed on day 297. On day 325, a diagnosis of peripheral T-cell lymphoma (PTCL) was made based on the T-cell receptor rearrangement seen in the tumor cells of his brain specimen. Therefore, on day 330, chemotherapy combined with high-dose methotrexate and cytarabine was initiated. A follow-up MRI on day 345 revealed that his CNS lesions had increased in size, suggesting that the chemotherapy regimen was ineffective, and he died of sepsis on day 348.

**Fig. 4:**
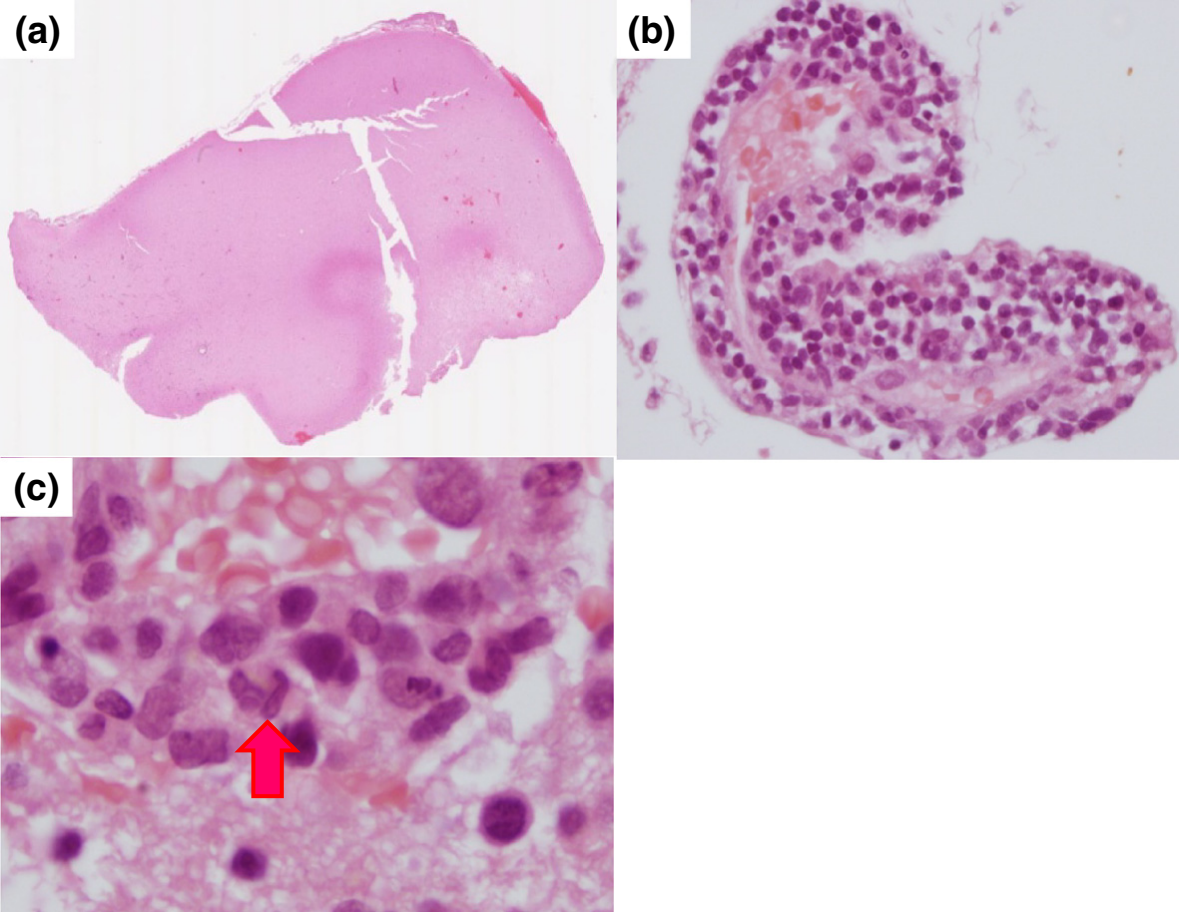
Brain biopsy findings. Brain tissue was stained with hematoxylin and eosin. **a** A brain lesion is distinguishable from the normal brain tissue. Magnification ×40. **b** Perivascular lymphocytic infiltrations is seen. Magnification ×100. **c** Some mitotic changes are observed (*arrow*). Magnification ×400

An autopsy revealed yellowish to brownish extended geographic lesions with softening, atrophy, and cavitation in the white matter of the frontal, temporal, and occipital lobes. Hepatomegaly (1910 g) and splenomegaly (205 g) were also observed. Microscopy of the cerebral white matter showed bilateral extended multiple liquefaction necroses (Fig. 4a). Infiltration of predominantly CD8(+) T cells undergoing dyskaryosis or mitosis was observed in the Virchow–Robin space (Fig. 4b, c), bone marrow, liver, and spleen. The rearrangements of the *TCR* gene were confirmed by PCR of a brain specimen. Hemophagocytosis was observed in the spleen and bone marrow. On immunohistochemistry, no cells were positive for herpes simplex virus or John Cunningham virus, and Epstein–Barr virus-encoded ribonucleic acid *in situ* hybridization was negative.

## Discussion

The patient presented here had a combination of several very rare diseases (DM, HLH, and CNS lesions). His condition was refractory, but it could be improved through intensive chemotherapy including etoposide (the modified HLH-2004 protocol).

Typically, most CNS lesions that appear acutely during immunosuppressive therapy are caused by infection. However, in this case, no infectious diseases were identified. Next, we suspected that the CNS lesions were caused by DM; however, his brain MRI findings revealed no evidence of vasculopathy or vasculitis, which are often seen in CNS lesions due to juvenile DM [7, 8]. Neuropsychiatric systemic lupus erythematosus was also excluded through blood and CSF examinations. Finally, we considered that the patterns of the CNS lesions on MRI closely resembled CNS lesions in patients with hereditary HLH who exhibit diffuse leptomeningeal and perivascular enhancement (corresponding to meningeal and perivascular infiltrations of histiocytes and lymphocytes), patchy areas of increased T2 signal intensity in the white matter of both cerebral hemispheres, and a diffuse parenchymal volume loss of the cerebrum and cerebellum [9]. Henter and Elinder described cases of progressive encephalopathy (termed CNS-HLH in their report) that were diagnosed on brain necropsy. The pathophysiology of CNS-HLH has been characterized as a perivascular infiltration of lymphocytes and histiocytes in the cerebral parenchyma [10].

CNS involvement is commonly seen in patients with hereditary HLH [11]. However, CNS involvement is less commonly seen in patients with autoimmune-associated HLH than in patients with malignancy-associated and viral infection-associated HLH. Gupta *et al*. reported that HLH was associated with rheumatic disease, malignancy, and viral/other complications by CNS disease in 14 %, 38 %, and 31 % of cases, respectively [12]. In our patient, natural killer cell activity was normal, and protein levels of Munc13-4, Munc18-2, syntaxin 11, and integrin αIIβ were normal; mutations in the genes coding for these proteins are associated with the onset of hereditary HLH [1]. Kim *et al*. reported that CNS involvement was associated with poor outcomes in patients with HLH and emphasized that the timely administration of chemotherapy (especially the early use of cyclosporine) was important for improving survival [13].

Yamashita *et al*. reported a case of HLH associated with DM complicated by CNS lesions; this case was identified on a postmortem examination [14]. Similar to our case, induction therapy, a combination of DEX pulse therapy and cyclophosphamide pulse therapy, and cyclosporine therapy was used in their patient. However, in contrast to Yamashita *et al*. [14] who used corticosteroids, cyclosporine, and cyclophosphamide, we used etoposide in accordance with the modified HLH-2004 protocol. We believe that the CNS lesions caused by HLH in our case improved owing to the early treatment based on the HLH-2004 protocol.

Haddad *et al*. have described the poor outcomes of CNS disease in patients with HLH. According to the results of their study, bone marrow transplantation (BMT) appears to be the only available treatment procedure capable of preventing HLH CNS disease progression and that can cure the patient when performed early after remission [11]. BMT was not performed after the recurrence of the CNS lesions in our case. In hindsight, considering the results, we should have performed BMT after the CNS lesions disappeared. The reason that we did not perform BMT was that there was no evidence for the effectiveness of BMT for HLH associated with autoimmune diseases complicated by CNS lesions, and those lesions had completely disappeared on MRI. Patients with DM are often known to have malignancies [5]. In our case, no malignancies were found on the PET-CT examination at the time of diagnosis of DM and HLH. We could not confirm the coexistence of PTCL on the day of admission because PET-CT examination and brain biopsy were not performed. Nevertheless, considering that the CNS lesions disappeared after the initial therapy and that PTCL cannot be controlled by immunochemotherapy according to the HLH-2004 protocol, we speculate that the initial CNS lesions were not from PTCL. If the CNS lesions had been derived from PTCL, it is unlikely that the CNS lesions would have disappeared with treatment following the HLH-2004 protocol. To the best of our knowledge, there is no report in the literature that PTCL complicated by CNS lesions could be cured by treatment following the HLH-2004 protocol. Therefore, we hypothesize that our patient experienced T-cell lymphoma either as an adverse effect of immunosuppressant therapy or due to chronic inflammation during his clinical course. 

## Conclusions

Our findings show that it is necessary to immediately administer additional intensive immunosuppressive therapies, such as those indicated in the HLH-2004protocol, including etoposide, to patients with corticosteroid-resistant acquired HLH due to autoimmune disease. In addition, a biopsy should be performed as early as possible for brain lesions of unknown origin. Patients with HLH with CNS lesions might require BMT to achieve good clinical outcomes.

## Abbreviations

BMT, bone marrow transplantation; CK, creatine kinase; CNS, central nervous system; CSF, cerebrospinal fluid; CT, computed tomography; DEX, dexamethasone; DM, dermatomyositis; HLH, hemophagocytic lymphohistiocytosis; mPSL, methylprednisolone; MRI, magnetic resonance imaging; PCR, polymerase chain reaction; PET-CT, positron emission tomography-computed tomography; PTCL, peripheral T-cell lymphoma
